# Editorial: Plant and human pathogen interactions: gaining insights into the impact of environmental and crop management factors

**DOI:** 10.3389/fpls.2024.1414227

**Published:** 2024-05-07

**Authors:** Cristián Jacob, Shirley A. Micallef, Maeli Melotto

**Affiliations:** ^1^ Departamento de Ciencias Vegetales, Facultad de Agronomía y Sistemas Naturales, Pontificia Universidad Católica de Chile, Santiago, Chile; ^2^ Department of Plant Science and Landscape Architecture, University of Maryland, College Park, MD, United States; ^3^ Centre for Food Safety and Security Systems, University of Maryland, College Park, MD, United States; ^4^ Department of Plant Sciences, University of California, Davis, Davis, CA, United States

**Keywords:** food safety, genotype diversity, water quality, microplastics, biocontrol, mulching

A significant portion of the world population struggles to access diets that support health, leading to micronutrient deficiencies, overweight, and obesity (FAO et al., 2023). To address these critical issues, global initiatives actively promote the production and consumption of fresh fruit and vegetables (FAO, 2017). These nutrient-rich foods are essential sources of vitamins, minerals, bioactive compounds, and dietary fiber crucial for enhancing overall health and reducing the risk of chronic, non-communicable diseases (Aune et al., 2017). However, a paradox arises as raw produce is increasingly linked to health risks due to outbreaks of human diseases (Aiyedun et al., 2021). These outbreaks significantly challenge the public health systems, agribusinesses, and consumer confidence, hindering efforts to strengthen the intake of nutritious fresh produce (Spalding et al., 2023).

Outbreak Investigations consistently indicate that the environmental presence of enteric pathogens is a major contributor of fresh produce contamination in the field (Bottichio et al., 2020; FDA, 2021). In agricultural settings, contamination risks arise from the use of low-quality water, improperly treated manure, and intruding animals (Matthews and Salvi, 2023). Research-based evidence strongly supports the notion that agronomic practices (*i*.*e*., soil management, irrigation systems, and harvest procedures) significantly influence the likelihood of crop contamination and pathogen persistence in edible plants (Lenzi et al., 2021). Furthermore, environmental factors such as temperature, relative humidity, insolation, wind, and rainfall may further impact crop safety (Belias et al., 2020).

Successful crop cultivation begins with the careful selection of cultivars possessing desired traits such as resistance to environmental stresses and overall yield potential (McAvoy and Ozores-Hampton, 2007). Intriguingly, research has revealed that intraspecific plant genetic variation significantly impacts the colonization of the phyllosphere by human bacterial pathogens such as *Salmonella enterica* (Jacob et al., 2024). These findings highlight the potential to select cultivars less conducive to human pathogen survival, while also elucidating the genetic bases underlying phenotypic variation. Conversely, the genetic diversity of pathogens profoundly shapes their fitness within agricultural environments. The study conducted by Wu et al. emphasized substantial variation in the inactivation dynamics of two surrogate viruses for human norovirus (NoV), Tulane virus (TV) and murine norovirus (MNV), on the surface of romaine lettuce leaves and in a sandy loam soil. In addition, differential colonization of lettuce leaf was detected among strains of various *S. enterica* serovars, attributed partly to their distinct abilities to utilize nutrients available in the apoplast and evade plant immune responses (Jacob et al., 2024). These studies expose the importance of investigating clinical, environmental, and outbreak isolates to identify high-risk strains or variants possessing traits with enhanced fitness to agricultural niches.

Soil management practices, including tillage, biological amendment, and crop rotation, are key to improve soil attributes such as structure, organic matter content, and microbiome composition (Welbaum, 2015). These practices play a crucial role in shaping the environmental conditions that impact the survival of human pathogens within agricultural soils. Using soils from three farms with different unamended soil types, Cook et al. observed that significant variation in the prevalence of *Listeria monocytogenes* and generic *E. coli* was associated with pH level. In addition, factors such as the farm’s geographical region and the presence of equipment and workers were also identified as influencing the likelihood of isolating *Listeria* spp. and *S. enterica* from the soil (Cook et al., 2023).

The presence of microplastics in soil poses a novel emerging threat to agriculture, as highlighted by Quilliam et al. Microplastics colonized by bacterial communities form the plastisphere, a niche that facilitates the survival of human pathogens in soil and their transfer to the rhizosphere, developing roots, and the surface of leaves and fruit (Quilliam et al., 2023). Microplastic accumulation in soils can be exacerbated by the increasing use of plastic mulching. This technology might also directly affect the dissemination of human pathogens throughout the field. Hopper et al. reported that the dispersal distance of *E. coli* strain TVS353 from inoculated rabbit manure to lettuce after natural or simulated rain events was significantly longer when lettuce beds were covered with various plastic mulches as compared to straw or bare soil.

Water can transfer human pathogens into agricultural fields and onto produce through a range of agronomic practices, including irrigation and agrochemical application (Matthews, 2023). Understanding the persistence capacity of human pathogens in water is essential for addressing food safety concerns. The study by Wu et al. revealed that human NoV surrogates exhibit remarkable stability in water. Notably, variations in water quality parameters did not affect the viral infectivity dynamics during incubation in water and after inoculations to soil and lettuce leaves (Wu et al., 2023). These findings uncover the potential adaptability of certain human pathogens for water environments and underscore the importance of analyzing water physicochemical parameters to assess the risk of pathogen survival.

The implementation of chemical, physical, and biological approaches to minimize human pathogens in food, water, and industrial surfaces is a key component of the multi-hurdle strategy aimed at ensuring the safety of fresh produce (Siddiqui, 2018). The study by Gollop et al. sheds light on the efficacy of seed treatments in reducing the prevalence of *Salmonella enterica* ser. Typhimurium in sprouted alfalfa seeds. The application of a food-derived *Bacillus* strain significantly reduced the pathogen populations, showcasing the potential of biological agents in enhancing food safety measures. Remarkably, this effect was further accentuated when the biological treatment was combined with calcium hypochlorite disinfection, emphasizing the synergistic potential of integrated approaches.

Farming encompasses a diverse array of agronomic practices that are essential for successful food production. Developing a comprehensive understanding of minimal-risk crop management practices across various environmental scenarios is crucial to ensure the sustainable production of fresh produce, prioritizing both human and environmental health. The articles in this Research Topic provide novel insights into the impact of environmental and crop management factors in the life of human pathogens in agricultural settings. Progress in this Research Topic is pivotal for updating and improving crop production policies and recommendations with science-based information aimed at enhancing food safety.

## Author contributions

CJ: Writing – original draft, Writing – review & editing. SM: Writing – original draft, Writing – review & editing. MM: Writing – original draft, Writing – review & editing.
